# Randomized placebo-controlled double-blind phase II study of zaltoprofen for patients with diffuse-type and unresectable localized tenosynovial giant cell tumors: a study protocol

**DOI:** 10.1186/s12891-019-2453-z

**Published:** 2019-02-09

**Authors:** Akihiko Takeuchi, Akihiro Nomura, Norio Yamamoto, Katsuhiro Hayashi, Kentaro Igarashi, Susumu Tandai, Akira Kawai, Akihiko Matsumine, Shinji Miwa, Yoshihiro Nishida, Tomoki Nakamura, Ryu Terauchi, Manabu Hoshi, Toshiyuki Kunisada, Makoto Endo, Kenichi Yoshimura, Toshinori Murayama, Hiroyuki Tsuchiya

**Affiliations:** 1 Department of Orthopaedic Surgery, Kanazawa University Graduate School of Medical Sciences, 13-1 Takara-machi, Kanazawa, 920-8641 Japan; 2 Innovative Clinical Research Center (iCREK), Kanazawa University Hospital, 13-1 Takara-machi, Kanazawa, 920-8641 Japan; 3 Department of Orthopaedic Surgery, Asahikawa Medical University, 2-1E Midorigaoka, Asahikawa, Hokkaido 078-8510 Japan; 4 Department of Musculoskeletal Oncology, National Cancer Center Hospital, 5-1-1 Tsukiji, Chuo-ku, Tokyo, 104-0045 Japan; 5 Department of Orthopaedics and Rehabilitation Medicine, Unit of Surgery, Division of Medicine, Faculty of Medical Sciences, University of Fukui, Fukui, 910-1104 Japan; 6 Department of Orthopaedic Surgery, Nagoya City University Graduate School of Medical Sciences, 1, Kawasumi, Mizuho-cho, Mizuho-ku, Nagoya, Aichi 467-8601 Japan; 7 Department of Orthopaedic Surgery, Nagoya University School of Medicine, 65 Tsurumai, Showa-ku, Nagoya, 466-8550 Japan; 8 Department of Orthopedic Surgery, Mie University School of Medicine, 2-174 Edobashi, Tsu, Mie 514-8507 Japan; 9 Department of Orthopaedic Surgery, Kyoto Prefectural University of Medicine, 465 Kajii-cho Hirokoji-agaru Kawaramachi-dori Kamigyo-ku, Kyoto, 602-8566 Japan; 10 Department of Orthopedic Surgery, Osaka City University Graduate School of Medicine, 1-4-3 Asahi-Machi, Abeno-ku, Osaka, 545-8585 Japan; 11 Department of Orthopedic Surgery, Okayama University Graduate School of Medicine, Dentistry, and Pharmaceutical Sciences, 2-5-1 Shikata-cho, Kita-ku, Okayama, 700-8558 Japan; 12 Department of Orthopaedic Surgery, Graduate School of Medical Sciences, Kyushu University, 3-1-1 Maidashi, Higashi-ku, Fukuoka, 812-8582 Japan

**Keywords:** Tenosynovial giant cell tumor, Zaltoprofen, Non-steroidal, Randomized control trial, Clinical trial

## Abstract

**Background:**

A tenosynovial giant cell tumor (TGCT) is a locally aggressive benign neoplasm arising from intra- or extra-articular tissue. Diffuse TGCT (D-TGCT) most commonly develops in the knee, followed by the hip, ankle, elbow, and shoulder. Surgical removal is the only effective treatment option for the patients. However, a local recurrence rate as high as 47% has been reported. Recently, we revealed that zaltoprofen, a nonsteroidal anti-inflammatory drug possessing the ability to activate peroxisome proliferator-activated receptor gamma (PPARγ), can inhibit the proliferation of TGCT stromal cells via PPARγ. PPARγ is a ligand-activated transcription factor that belongs to the nuclear hormone receptor superfamily. It plays an important role in the differentiation of adipocytes from precursor cells and exhibits antitumorigenic effects on certain malignancies. Therefore, we are conducting this investigator-initiated clinical trial to evaluate whether zaltoprofen is safe and effective for patients with D-TGCT or unresectable localized TGCT (L-TGCT).

**Methods:**

This study is a randomized, placebo-controlled, double-blind, multicenter trial to evaluate the safety and efficacy of zaltoprofen for patients with D-TGCT or L-TGCT. For the treatment group, zaltoprofen 480 mg/day will be administered for 48 weeks; the placebo group will receive similar dosages without zaltoprofen. Twenty participants in each group are needed in this trial (40 participants total). The primary outcome is the progression-free rate at 48 weeks after treatment administration. “Progression” is defined as any serious events (1. Repetitive joint swelling due to hemorrhage, 2. Joint range of motion limitation, 3. Invasion of adjacent cartilage or bone, 4. Severe joint space narrowing, 5. Increase in tumor size) requiring surgical interventions. We hypothesize that the zaltoprofen group will have a higher progression-free rate compared to that of the placebo group at 48 weeks.

**Discussion:**

This is the first study to evaluate the efficacy of zaltoprofen in patients with D-TGCT or unresectable L-TGCT. We believe that the results of this trial will validate a novel treatment option, zaltoprofen, to stabilize disease progression for TGCT patients.

**Trial registration:**

University Hospital Medical Information Network (UMIN) Clinical Trials Registry (UMIN000025901) registered on 4/01/2017.

## Background

A tenosynovial giant cell tumor (TGCT) is a locally aggressive benign neoplasm arising from intra- or extra-articular tissue. A localized extra-articular TGCT, also known as a giant cell tumor of the tendon sheath, commonly emerges from the smaller joints including the hand and ankle/foot. It is slight more predominant in women than in men [1] and an annual incidence has been reported as approximately one in 50,000 [2]. Diffuse TGCT (D-TGCT), is a synonymous with pigmented villonodular synovitis, grows in the large joint such as the knee, hip, ankle, elbow, and shoulder. and the recurrence rete is high despite the surgical removal. An incidence of it has been reported as approximately two cases per million annually with mostly younger than 40 years and a slight female predominance [3]. Surgical removal (open or arthroscopic synovectomy) is the only effective treatment, but the local recurrence rate has been reported to be 16 to 47% [4, 5]. It has been reported that TGCT growth is driven by overexpression of colony-stimulating factor 1 (CSF1) as a result of fusion of the CSF1 gene to the collagen type VI α3 (COL6A3) promoter in the t(1;2) translocation [6]. Therefore, systemic therapies targeting the CSF1/CSF1R axis have been tested in patients with locally advanced or relapsed D-TGCT [7].

Recently, we reported the case of a giant cell tumor of bone arising in the right distal femur, which demonstrated adipocyte lineage, strong expression of peroxisome proliferator-activated receptor gamma (PPARγ), and complete necrosis after taking zaltoprofen, a nonsteroidal anti-inflammatory drug (NSAID) for four weeks before biopsy [8]. PPARγ is a key transcriptional factor stimulates the adipocyte differentiation [9]. It also has the ability of antitumor activity by inhibiting tumor proliferation and invasion and through the induction of differentiation and apoptosis. PPARγ ligands including synthetic ligands such as thiazolidinedione (TZD) [10] and 15-deoxy-delta-12,14-prostaglandin J2 (15d-PGJ2) [11] have been investigated. Certain of NSAIDs, including indomethacin, play the role as direct ligands for PPARγ [12]. Zaltoprofen has been reported to induce apoptosis in rheumatoid synovial cells via the activation of PPARγ [13]. In some types of cancer, including liposarcoma [14], as well as colon cancer [15], breast cancer [16], and prostate cancer [17], the targeted-therapy for PPARγ has been tried. The long-term safety of zaltoprofen in the patients with rheumatoid arthritis was reported [18]. Based on those approaches, we analyzed the antitumor effect of zaltoprofen on primary cultured cells from TGCT, and zaltoprofen was found to inhibit their cell proliferation via activation of PPARγ [19].

We conducted a pilot study of zaltoprofen treatment for diffuse-type TGCTs arising in knee and ankle joints. This study included ten patients (6 knees and 4 ankles). Oral zaltoprofen (240 mg) was given daily for 48 weeks or until the disease was deemed progressive. Eight patients maintained stable disease at 48 weeks and one patient showed progressive disease at 72 weeks. At their request, surgery was performed for 3 patients with ankle D-TGCT at 12, 24, and 48 weeks, although all of them maintained stable disease. Since the zaltoprofen was well-tolerated and maintained stable disease [20], it could be a treatment option in patients with TGCT.

Herein, we describe an investigator-initiated clinical trial protocol to evaluate the efficacy and safety of zaltoprofen for patients with D-TGCT or unresectable localized TGCT (L-TGCT).

## Methods

### Overall study design

This study is a randomized, placebo-controlled, double-blind, multicenter trial, was designed by the investigators, and was accepted by the Pharmaceuticals and Medical Devices Agency (PMDA). The Center for Clinical Trials, Japan Medical Association (JMACCT) funded this trial (JMA-IIA00284). The trial network consisted of a lead site at Innovative Clinical Research Center, Kanazawa University (iCREK) (Kanazawa, Japan) and ten additional sites in Japan; Asahikawa Medical University Hospital; National Cancer Center Hospital; Fukui University Hospital; Nagoya City University Hospital; Nagoya University Hospital; Mie University Hospital; University Hospital, Kyoto Prefectural University of Medicine; Osaka City University Hospital; Okayama University Hospital; and Kyusyu University Hospital. The aim of this study is to evaluate the safety and efficacy of zaltoprofen for patients with D-TGCT or L-TGCT. For the treatment group, zaltoprofen 480 mg/day will be administered for 48 weeks. The overall follow-up schedule is shown in Table 1.1.

**Table 1:** Assessment and evaluation schedule of this study

Assessments		Screening tests	Drug admin	Study period (weeks)												Joint puncture *^4^	CR/PR *^5^	W/D *^6^	4 weeks after final drug admin	F/U after trial
				4	8	12	16	20	24	28	32	36	40	44	48					
	Informed consent	x																		
	Randomization	x																		
	Study drug admin/ adherence check																	x		
Physical exam	Clinical findings	x	x *^3^	x	x	x	x	x	x	x	x	x	x	x	x			x	x	o
	Joint findings	x	x *^3^	x	x	x	x	x	x	x	x	x	x	x	x	x	x	o *^7^	x	o
	Joint function	x	x *^3^	x	x	x	x	x	x	x	x	x	x	x	x	x	x	o *^7^	x	o
	VAS scale	x	x *^3^	x	x	x	x	x	x	x	x	x	x	x	x	x	x	o *^7^	x	o
	Joint fluid findings															x				
	Height	x	x																	
	Weight	x	x			x			x			x			x			x	x	
	Vital signs	x	x	x	x	x	x	x	x	x	x	x	x	x	x				x	
Imaging	CT or MRI	x *^1^				x			x			x			x		x	o *^7^	o *^8^	o
	FDG-PET	x *^1^							x						x					
	X-ray	x *^1^				x			x			x			x		x	o *^7^	o *^8^	o
	12-lead ECG	x *^1^																	x	
Clinical exam	Blood test & urinalysis	x		x		x			x			x			x			x	x	
	Pregnancy test	o *^2^																		
	F/U evaluation																	x	x	o
	Adverse event																	x		
	Concomitant medications																	x		

*x* mandatory, *o* if required, *exam* examination, *VAS* visual analog scale, *CT* computed tomography, *MRI* magnetic resonance imaging, *FGD-PET* ^18^F-fluorodeoxyglucose- positron emission tomography, *ECG* electrocardiography, *F/U* follow up, *admin* administration, *CR* complete response, *PR* partial response, *W/D* withdrawal

*^1^: Can be used in 28 days before registration, *^2^: Only for women, *^3^: Must be evaluated before drug administration, *^4^: Must be evaluated before joint puncture, *^5^: Must be evaluated in 8 weeks ± 4 weeks after CR or PR, *^6^: Must be evaluated in ±7 days of final drug administration and before initiating post-treatments *^7^: Not necessary if disease progression is detected or post-treatments are initiated before “the timing of drug withdrawal” *^8^: Not necessary if disease progression is detected or post-treatments are initiated before “28 days after final drug administration” *^9^: Must be done until detection of progression or post-treatment initiation

**Fig. 1:**
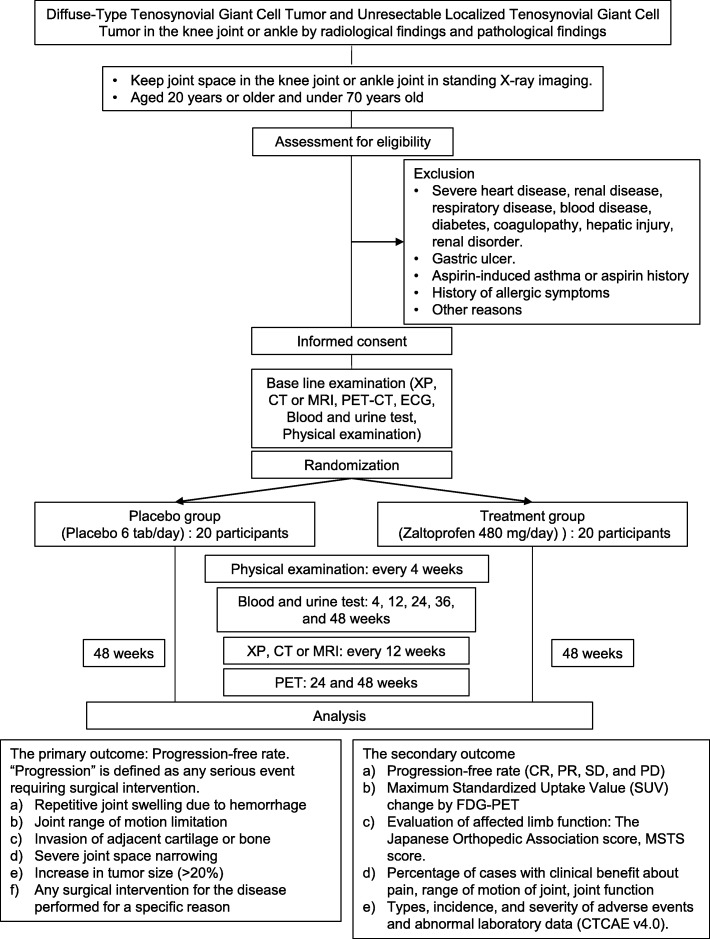
Scheme of this study protocol. CT, computed tomography; ECG, electrocardiogram; MRI, magnetic resonance imaging; PET, positron emission tomography; XP, X-ray photograph; CTCAE, common terminology criteria for adverse events; CR, complete response; PR, partial response; SD, stable disease; PD, progressive disease; FDG, ^18^F-fluorodeoxyglucose; MSTS, Musculoskeletal Tumor Society

The primary outcome of this study is the progression-free rate at 48 weeks after treatment administration. “Progression” is defined as any serious event requiring surgical intervention (Fig. 1). We hypothesize that the zaltoprofen group will have a higher progression-free rate compared to that of the placebo group at 48 weeks.

We are conducting this trial in accordance with the Declaration of Helsinki, the Ethical Guidelines for Medical and Health Research Involving Human Subjects, and all other applicable Japanese laws and guidelines. The protocol was approved by the Institutional Review Board (IRB) at Kanazawa University Hospital and each participating hospital. This study is registered at the University Hospital Medical Information Network (UMIN) Clinical Trials Registry (UMIN000025901).

### Study participants

We are recruiting D-TGCT and L-TGCT patients from April 2017 until March 2020, or until the enrollments are completed. We are including patients who meet all of the inclusion criteria (Table 2) (Fig. 1), but are excluding those who possess any of the exclusion criteria (Table 3). We are obtaining written informed consent from all the trial participants. These consent forms were also approved by the IRB. Each participant must understand the contents of the consent form before one’s acceptance. Each form must be dated and signed by both the participant and trial investigators. We also inform the participants that their medical treatment will not be affected if they refuse to participate in this trial. The consent forms are stored at each institution. The participants can drop out of the trial at any time.

**Table 2:** Inclusion criteria

We include patients with all of the following criteria:	
1)	Patients who have been made aware of the research purpose, interests, and disadvantages of the clinical trial before starting the examination - They understand this, and written informed consent is obtained. No substitution is allowed.
2)	Patients who are diagnosed with diffuse-type tenosynovial giant cell tumor (pigmented chorionic synovitis) or unresectable localized tenosynovial giant cell tumor occurring in the knee joint or ankle by radiological and pathological findings
3)	Patient with measurable lesion based on RECIST^a^ v1.1 with at least one knee or ankle joint
4)	Patients who keep joint space in the knee or ankle joint in standing X-ray imaging
5)	Patients aged 20 years or older but less than 70 years old at the time of acquisition
6)	In the case of a pregnant woman, a patient whose pregnancy test, to be conducted during the screening period, is negative

^a^*RECIST* response evaluation criteria in solid tumors

**Table 3:** Exclusion criteria

We exclude patients with any of the following criteria:	
1)	Patients with severe heart disease, renal disease, respiratory disease, blood disease, diabetes, coagulopathy, hepatic injury, or renal disorder
2)	Patients with gastric ulcer
3)	Patient with aspirin-induced asthma or history of aspirin
4)	Patients who have had a history of allergic symptoms such as itching and rash, or taking zaltoprofen (Soleton tablet 80 mg, Peon tablet 80 mg, etc.) previously
5)	Hypersensitivity to additives in zaltoprofen (lactose hydrate, corn starch, cellulose, silicic anhydride, hydroxypropyl cellulose, carmellose Ca, stearic acid Mg, polysorbate 80, hypromellose, titanium oxide, talc, carnauba wax) or patient with a history of hypersensitivity
6)	Patients who are administered any of the following agents within 14 days prior to enrollment: tyrosine kinase inhibitors, nonsteroidal anti-inflammatory drugs, thiazolidine derivatives, and drugs with a thiazolysine ring
7)	Patients who have an active range of motion limitation of > 20% with respect to the healthy joint
8)	Patients whose joint space has disappeared by standing position X-ray photography
9)	Patients who are inappropriate for examinations, such as MRI and PET^a^
10)	Patients who are pregnant or lactating or patients who do not agree to contraception from the final administration of the study drug to 90 days thereafter
11)	Patients who have difficulty taking oral medications
12)	Patients who were using other investigational drugs or using the investigational drug within 3 months prior to the study drug administration
13)	A patient whose investigator or clinical trial doctor judged him/her unsuitable for participation in this trial due to other reasons

^a^*MRI* magnetic resonance imaging, *PET* positron emission tomography

Please ensure that this wording is correct

### Randomization

We perform randomization at the time of trial registration (Fig. 1). One of the trial staff allocates participants to the two arms (1:1), one group receiving zaltoprofen and the other placebo. Randomization is performed by using a computer-generated random sequence with stratification for the size of tumor lesion and tumor location (i.e., knee or foot joints).

### Intervention and placebo

For the treatment (intervention) group, we prescribe 2 oral tablets of zaltoprofen (80 mg per tablet), 3 times daily with approximately one cup of water (~ 150 mL). For the placebo group, we prescribe 2 oral placebo tablets, 3 times daily with the same volume of water (Fig. 1). For both groups, we also prescribe a gastric mucosal protective agent in regular dosage during the study period.

### Outcomes

The primary outcome is the progression-free rate at 48 weeks after drug administration. Our definition of progression (exacerbation) is any serious event requiring surgical intervention. Exacerbation is indicated if a patient meets one of the following criteria:

i). The joint circumference is increased by 2 cm or more with respect to the baseline (knee: 1 cm above patella; ankle joint: determined using the figure-eight method). If there is fluid accumulation, it is examined for the presence or absence of hematoma. An increase in joint circumference due simply to edema is not considered exacerbation.
ii). The range of motion of the joint (i.e., active motion) is reduced by 20% or more with respect to the baseline (calculated by averaging three measurements with a goniometer).
( i) or ii) must be detected in 2 consecutive evaluation periods conducted every 4 weeks)
iii) An invasion of 5 mm or more of bone / cartilage erosion or a new lesion of bone / cartilage erosion of 5 mm or more, compared with that of baseline, is detected by computed tomography (CT) or magnetic resonance imaging (MRI).
iv) An X-ray in the standing position shows the disappearance of joint space.
v) An increase in the target lesion by 20% or more is determined by the response evaluation criteria in solid tumors (RECIST).
( iii), iv) or v) must be detected once in any evaluation period conducted every 12 weeks).
vi) Any surgical intervention for the disease that is performed for a specific reason.

We also evaluate secondary outcomes as follows:

- Progression-free rate (24 weeks and 48 weeks): Percentage of cases of complete response (CR), partial response (PR), and stable disease (SD, i.e., when CR and PR are unchanged for more than 4 weeks).
- Maximum Standardized Uptake Value (SUV) change by ^18^F-fluorodeoxyglucose- positron emission tomography (FDG-PET).
- Evaluation of affected limb function (baseline, 24 weeks, and 48 weeks): The Japanese Orthopedic Association score and Musculoskeletal Tumor Society (MSTS) score.
- Percentage of cases in which clinical benefit regarding pain, range of joint motion, and joint function compared with that of baseline is observed at 24 weeks and 48 weeks (judgment by investigators or clinical trial physicians).
- The types, incidence, and severity of adverse events and abnormal laboratory data (graded using common terminology criteria for adverse events (CTCAE) v4.0) will be assessed, evaluating the severity and relationship with the investigational drug (Fig. 1).

### Safety assessment

The investigator of each institutions ensure that adequate medical care is provided to a subject for any adverse events, including clinically significant laboratory values, related to the trial. All serious adverse events (SAEs) will be reported immediately to IRB of their institution and to the PI. The unblinding is permissible in case of SAEs. The PI will report to the IRB at Kanazawa University and PMDA according to the international conference on harmonisation (ICH) guideline-E6 and E2A (https://www.ich.org/home.html). The PI also will ensure the SAEs to all investigator and institution. If necessary, the independent data monitoring committee (IDMC) will be established.

### Follow-up schedule

The overall follow-up schedule in this trial is shown in Table 1. Follow-up visits are conducted at each institution. We expect that regular follow-up visits will be scheduled every 4 weeks (± 7 days) to check the patients’ vital signs and to perform physical examinations. We will also evaluate disease progression by diagnostic imaging every 12 weeks, using X-ray, CT/MRI, or PDG-PET as scheduled. Blood tests are scheduled to be performed at 4 weeks and every 12 weeks thereafter (at the same time as diagnostic imaging). We will evaluate the primary outcome (i.e., the progression-free rate) at 48 weeks (Fig. 1). Adverse events and concomitant medications will be recorded through the trial.

### Sample size

We consider the progression-free rate at 48 weeks after treatment administration as the main outcome. We estimated the progression-free rates in the zaltoprofen (treatment) and placebo groups to be 80 and 30%, respectively. In the sample size calculation, we used the log-rank test to determine that 20 participants per group would be required for a 95% confidence interval and power of 90%. Thus, a total of 40 participants will be needed in this trial.

### Statistical analysis

The outcomes between the zaltoprofen treatment group and placebo group will be compared. Baseline characteristics will be described by means and standard deviations, or medians and quantiles (for continuous variables), and proportions (for categorical variables). The primary outcome will be analyzed based on the full analysis set (excluding participants who violate inclusion/exclusion criteria or do not take the study drugs), and will be compared between the groups at 48 weeks using the log-rank test. The secondary outcomes will also be compared between the groups at 24 and 48 weeks using *t*-tests, Mann-Whitney U tests, or Fisher’s exact tests. *P*-values less than 0.05 will be considered significant.

## Discussion

To the best of our knowledge, this is the first evaluation of the efficacy of zaltoprofen for patients with diffuse-type and unresectable localized TGCT by a randomized placebo-controlled double-blind phase II investigator-initiated study.

TGCT progresses slowly [21]. However, it causes local destruction with invasion of the adjacent joint cartilage [22], and more severe symptoms such as joint pain, limited range of motion, swelling, erythema, and hemorrhagic effusion [23]. Excision by arthroscopic or open synovectomy is recommended, but complete removal is sometimes difficult to achieve due to the wide spread of the growth, resulting in high local recurrence [4, 5]. In addition, total joint replacement is necessary in the case of severe joint destruction due to tumor progression [24].

Based on our pilot study [20], we hypothesized that zaltoprofen treatment would produce a higher progression-free rate than that of placebo at 48 weeks. Moreover, we considered that the benefit of zaltoprofen is to avoid serious events, such as repetitive hemorrhage effusion, limitation of range of motion, adjacent joint cartilage destruction, and tumor enlargement, which require surgical interventions. Therefore, we defined the criteria of exacerbation in each serious event: 1) An increase in joint circumference by 2 cm or more with respect to the baseline measurement by hematoma is used to reflect repetitive hemorrhage in the joint, which risks joint cartilage destruction by inflammation [25]. 2) A reduced range of motion of the joint (i.e., active motion) by 20% or more with respect to the baseline value is used because the limited range of motion represents joint destruction and 20% reduction is the worst Ogilvie-Harris score [26]. 3) An invasion of 5 mm or more of bone / cartilage erosion or new lesions of bone /cartilage erosion of 5 mm or more compared with that of baseline by CT or MRI is used because bone invasion is responsible for joint destruction and MRI can detect 5 mm of invaded tumor [27]. 4) The disappearance of joint space detected by X-ray photography in the standing position is used because severe joint space narrowing is the worst grade of the Kellgren and Lawrence (KL) grading system [28]. and 5) An increase in the target lesion size by 20% or more by RECIST is used because a ≥ 20% increase in the sum of the diameters of the target lesions is defined as progressive disease (PD) by the RECIST criteria [29].

We believe that the results of this trial can conclusively identify zaltoprofen as a novel treatment strategy to stabilize disease progression for patients with D-TGCT or unresectable L-TGCT. The recruiting of eligible patients has been ongoing since April, 2017.

## Abbreviations

15d-PGJ2: 15-deoxy-delta-12,14-prostaglandin J2
COL6A3: Collagen type VI α3
CR: Complete response
CSF1: Colony-stimulating factor 1
CT: Computed tomography
CTCAE: Common terminology criteria for adverse events
D-TGCT: Diffuse tenosynovial giant cell tumor
FDG-PET: ^18^F-fluorodeoxyglucose- positron emission tomography
ICH: International conference on harmonisation
IRB: Institutional Review Board
JMACCT: The Center for Clinical Trials, Japan Medical Association
KL grading: Kellgren and Lawrence grading
L-TGCT: Localized tenosynovial giant cell tumor
MHLW: Ministry of Health, Labor and Welfare
MRI: Magnetic resonance imaging
MSTS: Musculoskeletal Tumor Society
PD: Progressive disease
PMDA: Pharmaceuticals and Medical Devices Agency
PPARγ: Peroxisome proliferator-activated receptor gamma
PR: Partial response
RECIST: Response evaluation criteria in solid tumors
SAEs: Serious adverse events
SD: Stable disease
SUV: Standardized uptake value
TGCT: Tenosynovial giant cell tumor
TZD: Thiazolidinedione
UMIN: University Hospital Medical Information Network
USAR: Suspected, unexpected, serious adverse reaction

## References

[1] Jones FE, Soule EH, Coventry MB (1969). Fibrous xanthoma of synovium (giant-cell tumor of tendon sheath, pigmented nodular synovitis). A study of one hundred and eighteen cases. J Bone Joint Surg Am.

[2] Monaghan H, Salter DM (2001). Al-Nafussi a. Giant cell tumour of tendon sheath (localised nodular tenosynovitis): clinicopathological features of 71 cases. J Clin Pathol.

[3] Fletcher C, Bridge J, Hogendoom P, Mertens F, de Saint Aubain Somerhausen N, van de Rijn M (2013). Tenosynovial giant cell tumor, diffuse type. WHO classification of tumours of soft tissue and b; World Health Organization; International Agency for Research on Cancer.

[4] Ma X, Shi G, Xia C, Liu H, He J, Jin W (2013). Pigmented villonodular synovitis: a retrospective study of seventy five cases (eighty one joints). Int Orthop.

[5] Sharma V, Cheng EY (2009). Outcomes after excision of pigmented villonodular synovitis of the knee. Clin Orthop Relat Res.

[6] West RB, Rubin BP, Miller MA, Subramanian S, Kaygusuz G, Montgomery K (2006). A landscape effect in tenosynovial giant-cell tumor from activation of CSF1 expression by a translocation in a minority of tumor cells. Proc Natl Acad Sci U S A.

[7] Brahmi M, Vinceneux A, Cassier PA (2016). Current systemic treatment options for Tenosynovial Giant cell tumor/pigmented Villonodular synovitis: targeting the CSF1/CSF1R Axis. Curr Treat Options in Oncol.

[8] Takeuchi A, Yamamoto N, Nishida H, Kimura H, Ikeda H, Tsuchiya H (2013). Complete necrosis of a giant cell tumor with high expression of PPARγ: a case report. Anticancer Res.

[9] Rosen ED, Hsu CH, Wang X, Sakai S, Freeman MW, Gonzalez FJ, et al. C/EBPalpha induces adipogenesis through PPARgamma: a unified pathway. Genes Dev. 2002;16:22–6. 10.1101/gad.948702 PMC155311 11782441

[10] Lehmann JM, Moore LB, Smith-Oliver TA, Wilkison WO, Willson TM, Kliewer SA (1995). An antidiabetic thiazolidinedione is a high affinity ligand for peroxisome proliferator-activated receptor gamma (PPAR gamma). J Biol Chem.

[11] Forman BM, Tontonoz P, Chen J, Brun RP, Spiegelman BM, Evans RM (1995). 15-deoxy-Δ12,14-prostaglandin J2is a ligand for the adipocyte determination factor PPARγ. Cell.

[12] Lehmann J, Lenhard J (1997). Peroxisome proliferator-activated receptors α and γ are activated by indomethacin and other non-steroidal anti-inflammatory drugs. J Biol.

[13] Yamazaki R, Kusunoki N, Matsuzaki T, Hashimoto S, Kawai S (2002). Nonsteroidal anti-inflammatory drugs induce apoptosis in association with activation of peroxisome proliferator-activated receptor gamma in rheumatoid synovial cells. J Pharmacol Exp Ther.

[14] Demetri GD, Fletcher CD, Mueller E, Sarraf P, Naujoks R, Campbell N (1999). Induction of solid tumor differentiation by the peroxisome proliferator-activated receptor-gamma ligand troglitazone in patients with liposarcoma. Proc Natl Acad Sci U S A.

[15] Kulke MH, Demetri GD, Sharpless NE, Ryan DP, Shivdasani R, Clark JS (2002). A phase II study of troglitazone, an activator of the PPARgamma receptor, in patients with chemotherapy-resistant metastatic colorectal cancer. Cancer J.

[16] Burstein HJ, Demetri GD, Mueller E, Sarraf P, Spiegelman BM, Winer EP (2003). Use of the peroxisome proliferator-activated receptor (PPAR) gamma ligand troglitazone as treatment for refractory breast cancer: a phase II study. Breast Cancer Res Treat.

[17] Hisatake J-II, Ikezoe T, Carey M, Holden S, Tomoyasu S, Koeffler HPP (2000). Down-regulation of prostate-specific antigen expression by ligands for peroxisome proliferator-activated receptor γ in human prostate cancer. Cancer Res.

[18] Hatori M, Kokubun S (1998). The long-term efficacy and tolerability of the new anti-inflammatory agent Zaltoprofen in rheumatoid arthritis. Curr Med Res Opin.

[19] Takeuchi A, Yamamoto N, Shirai T, Nihida H, Hayashi K, Kimura H (2015). Effect of Zaltoprofen for pigmented Villonodular synovitis cells with PPARγ activation. Proceedings of AAOS meeting Las Vegas, NV.

[20] Takeuchi A, Yamamoto N, Hayashi K, Miwa S, Inatani H, Aoki Y (2017). Feasibility as novel treatment of Zaltoprofen for diffuse-type tenosynovial giant cell tumor arising in knee and ankle joint: a pilot study. Proceedings of ISOLS meeting Kanazawa, Japan.

[21] Nassar WAM, Bassiony AA, HA E (2009). Treatment of diffuse pigmented villonodular synovitis of the knee with combined surgical and radiosynovectomy. HSS J.

[22] Chen K, Ren Q, Han XR, Zhang XN, Wei B, Bai XZ (2016). Imatinib mesylate induces mitochondria-dependent apoptosis and inhibits invasion of human pigmented villonodular synovitis fibroblast-like synovial cells. Oncol Rep.

[23] Aboulafia A, Kaplan L, Jelinek J, Benevenia J, Monson D (1996). Neuropathy secondary to pigmented villonodular synovitis of the hip. Clin Orthop Relat Res.

[24] Hamlin BR, Duffy GP, Trousdale RT, Morrey BF (1998). Total knee arthroplasty in patients who have pigmented villonodular synovitis. J Bone Joint Surg Am.

[25] Roosendaal G, Vianen ME, Wenting MJ, van Rinsum AC, van den Berg HM, Lafeber FP (1998). Iron deposits and catabolic properties of synovial tissue from patients with haemophilia. J Bone Joint Surg Br.

[26] Ogilvie-Harris DJ, McLean J, Zarnett ME (1992). Pigmented villonodular synovitis of the knee. The results of total arthroscopic synovectomy, partial, arthroscopic synovectomy, and arthroscopic local excision. J Bone Joint Surg Am.

[27] Schmidt GP, Reiser MF, Baur-Melnyk A (2007). Whole-body imaging of the musculoskeletal system: the value of MR imaging. Skelet Radiol.

[28] Kellgren JH, Lawrence JS (1957). Radiological assessment of osteo-arthrosis. Ann Rheum Dis.

[29] Eisenhauer EA, Therasse P, Bogaerts J, Schwartz LH, Sargent D, Ford R (2009). New response evaluation criteria in solid tumours: revised RECIST guideline (version 1.1). Eur J Cancer.

